# City structure shapes directional resettlement flows in Australia

**DOI:** 10.1038/s41598-020-65208-5

**Published:** 2020-05-19

**Authors:** Bohdan Slavko, Kirill Glavatskiy, Mikhail Prokopenko

**Affiliations:** Centre for Complex Systems, The University of Sydney, Sydney, NSW 2006 Australia

**Keywords:** Sustainability, Applied mathematics, Thermodynamics

## Abstract

Modern urban science views differences in attractiveness of residential suburbs as the main driver of resettlement within a city. In particular, certain suburbs may attract residents due to lower commute costs, and this is believed to lead to compactification of a city, with highly populated central business district and sprawled suburbia. In this paper we assess residential resettlement patterns in Australian capital cities by analyzing the 2011 and 2016 Australian Census data. Rather than explicitly defining a residential attractiveness of each suburb in subjective terms, we introduce and calibrate a model which quantifies the intra-city migration flows in terms of the attractiveness potentials (and their differences), inferring these from the data. We discover that, despite the existence of well-known static agglomeration patterns favouring central districts over the suburbia, the dynamic flows that shape the intra-city migration over the last decade reveal the preference directed away from the central districts with a high density of jobs and population, towards the less populated suburbs on the periphery. Furthermore, we discover that the relocation distance of such resettlement flows plays a vital role, and explains a significant part of the variation in migration flows: the resettlement flow markedly decreases with the relocation distance. Finally, we propose a conjecture that these directional resettlement flows are explained by the cities’ structure, with monocentric cities exhibiting outward flows with much higher reluctance to long-distance relocation. This conjecture is verified across the major Australian capitals: both monocentric (Sydney, Melbourne, Brisbane, Adelaide, Perth, Hobart) and polycentric (Darwin and Canberra).

## Introduction

The modern world undergoes rapid urbanisation^1^. Growing complexity of cities demands new fundamental understanding and rigorous models of the urban dynamics, including diverse and transient intra-urban migration patterns. At the same time, the increasing availability of large-scale spatiotemporal data (e.g., from censuses or surveys) provides an additional impetus for quantitative modelling of urban development and related dynamics^2–4^. A consistent modeling framework for settlement formation does not yet exist^2^, despite an abundance of stable empirical patterns^5–12^ and theoretical insights^13–22^ that can potentially improve our understanding of how cities grow and self-organise.

Contemporary cities are shaped by the long process of settlement formation and evolution which involves multiple types of migration and resettlement. In social sciences it is common to investigate population migration in the context of individual motivation and social consequences^23–25^. However, heterogeneous preferences of individual residents cannot be easily integrated into their aggregate migration flows. The latter is a collective phenomenon which we believe is determined mainly by the spatial city structure^2^. Furthermore, such migration patterns are usually persistent in time. In particular, we observe strong correlation between migration flows in two different periods of 2006–2011 and 2011–2016 (see Fig. 6 in the Supplementary Material). Focusing on a small number of macroscopic parameters (such as migration flow) instead of a large number of microscopic degrees of freedom (individual human choices) is one of the underlying motivations of this study, developed from the perspective of sociophysics^26–31^.

Although static properties of modern cities have been studied quite extensively, the spatial evolution of cities over time remains a subject of vigorous research. Often the dynamics of evolution is typically assumed to be determined by the current static structure. In particular, the compact structure of modern cities can be explained by the transportation cost minimisation^18,32^, so that the already dense central business districts attract even more jobs and more residents, while the sparsely populated city’s periphery is being diluted. An actual verification of this division is subject to data availability. For example, various statistics of resettlement processes may be traced on the national level using census data, but typically, detailed information is not collected. In particular, the US^33^ or Canadian^34^ census datasets do not contain records of previous places of residence, and instead merely include relocation events — as a result, this prevents tracking intra-urban migration flows and studying their influence on the spatial structure of the cities. In contrast, the Australian Census^35^ does collect such information, which makes it a source of unique data for dynamic analysis. Thus, a detailed analysis of the Australian Census data can provide insights of how the static agglomeration patterns affect the dynamic evolution of a city.

The notion of subjective suburb’s attractiveness is often considered in the context of identifying the city structure^18,20,27,36^. We introduce a model comprising the concept of a *revealed attractiveness* potential that drives intra-urban migration. This potential absorbs all characteristics of a suburb, which indicate to what extent people are statistically attracted to it — this potential hence affects the aggregate relocation flows. These characteristics may include the distance to the centers of business or retail activity^37,38^, the public transport accessibility^39^, the quality of local infrastructure (schools and other local services)^36,40^, the housing affordability^37,41^, and perhaps, other publicly known but not explicitly defined subjective factors. Our model treats the attractiveness as a revealed quantitative characteristic of a suburb, inferring it from data, rather than being based on a particular subjective characterization.

**Figure 1:**
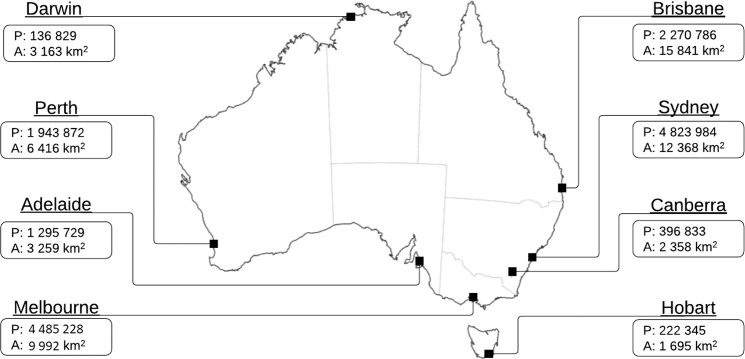
Australian capital cities: location on the map, population (P), and area (A), according to 2016 Census.

Using our resettlement model, we analyze intra-urban migration flows in the Australian capital cities, represented by the corresponding Greater Capital Areas (GCA): Sydney, Melbourne, Brisbane, Adelaide, Perth, Hobart, Darwin, and Canberra (Fig. 1). These cities are the major centres of business, economic and cultural activities across Australian states and territories, and are well distanced from each other. Furthermore, these cities vary in trajectories of their historical development, ranging from short periods of centralized planning to long intervals of self-organized evolution. The general diversity observed across major urban centres is expected to be reflected in patterns of modern urban dynamics^42,43^ and, in particular, in patterns of the resettlement dynamics.

We use the latest Census data to estimate the spatial distribution of revealed attractiveness and its contribution to the migration flows. As a result, we discover that the role of the suburbs’ attractiveness is opposite to what may be expected by a naive perspective: the migration flows are directed away from the central districts with a high population density and infrastructure accessibility, towards sparsely populated suburbs (typically located at the city’s outskirts). In addition, the attractiveness of suburbs alone does not allow one to predict the resettlement flows with an adequate precision. In particular, we observe that, to a large extent, the migration flows are determined by the relocation distance: the short-distance relocations occur much more often than the long-distance ones.

**Figure 2:**
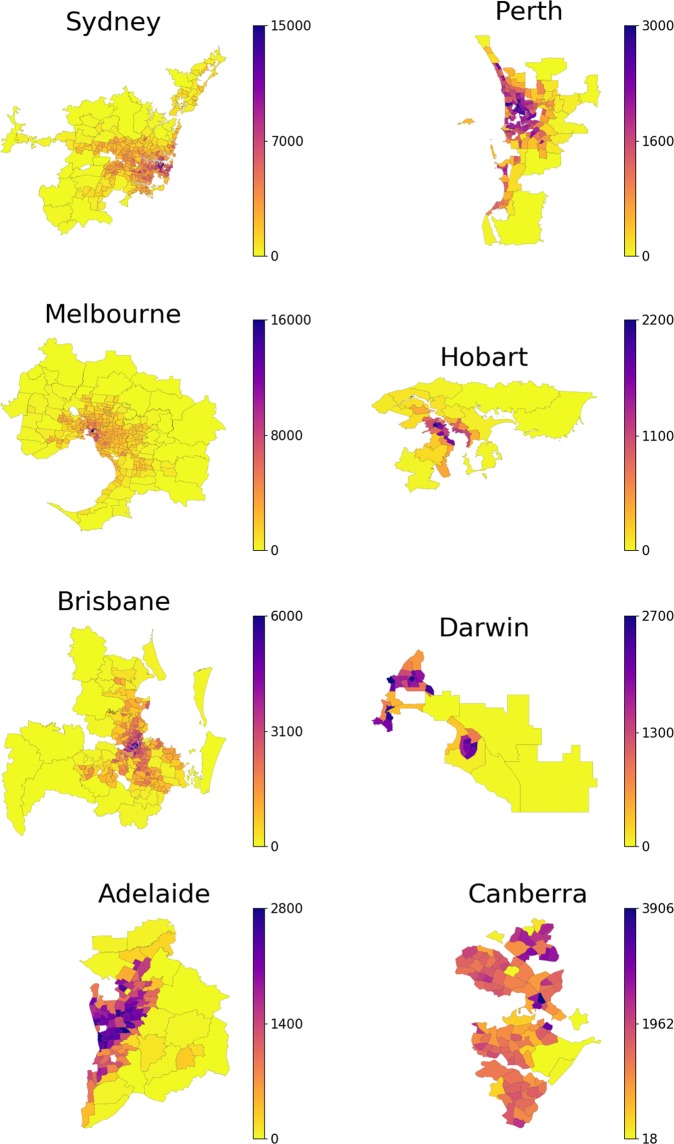
Urban sprawl of GCA, according to 2016 Census. Yellow color corresponds to the lowest population, while blue color corresponds to the highest population. The bar on the right encodes the value of the density measured in the amount of people per km^2^. Note that the density range and therefore the color correspondence are different for each GCA. The suburbs with the population density less than 10 people/km^2^ are not shown.

Finally, we observe and quantify a specific relationship between the dynamic migration patterns and the static city structure, defined in terms of its polycentricity (as illustrated by e.g. Fig. 2). Namely, six out of eight capital cities (Sydney, Melbourne, Brisbane, Adelaide, Perth, Hobart) are monocentric, and at the same time exhibit a high *reluctance to long-distance relocations*, with a clear center-to-periphery migration flow. In contrast, two out of eight capital cities (Darwin and Canberra) are non-monocentric, while exhibiting a low reluctance to long-distance relocations without a well-defined flow direction. While it is hard to make a definite conclusion based on small number of cities, we make a conjecture: not only does the human resettlement affect the spatial structure of cities, but the city structure itself may also affect the intra-urban migration. A proper verification of this conjecture is subject to future research and data availability.

## Results

We analyze residential migration using thee related hypotheses, which are referred here as the baseline hypothesis, the extended hypothesis, and the spatial hypothesis. The baseline hypothesis investigates the sole effect of the attractiveness potentials *per se*. The extended hypothesis augments the baseline with the relocation distance dependence. Finally, the spatial hypothesis focuses on the relocation distance alone.

According to all hypotheses the migration flow ${T}_{ik}$ (amount of relocated people per unit of time) from suburb *i* to suburb *k* is proportional to the populations ${P}_{i}$ and ${P}_{k}$ of either suburb, as well as to the migration driving force $exp({f}_{ik})$:

1 $${T}_{ik}=\mu \,{P}_{i}\,{P}_{k}\,{e}^{{f}_{ik}},$$

where $\mu$ is a positive normalizing constant. In particular, we fit Census data on migration flows ${T}_{ik}$ to the model equations and judge on the validity of each hypothesis based on the goodness of fit and on the number of fitting parameters, which are summarized in Table 1.

**Table 1:** Calibration details of migration models (2)–(4) fitted to the 2011–2016 data.

GCA	# of parameters			${{\boldsymbol{R}}}^{2}$		
	baseline	extended	spatial	baseline	extended	spatial
Sydney	313	314	2	0.04	0.68	0.59
Melbourne	310	311	2	0.04	0.70	0.58
Brisbane	237	238	2	0.06	0.68	0.64
Adelaide	111	112	2	0.13	0.76	0.72
Perth	174	175	2	0.13	0.72	0.58
Hobart	36	37	2	0.16	0.65	0.64
Darwin	45	46	2	0.36	0.62	0.45
Canberra	132	133	2	0.36	0.78	0.62

We observe, that the baseline hypothesis (2) provides the worst fit, both in terms of the coefficient of determination and the number of parameters. In contrast, the extended hypothesis (3) uses only one parameter more, but provides the best fit in terms of the coefficient of determinations. Finally, the spatial hypothesis (4) isolates the differences between the extended and the baseline hypothesis and uses 2 parameters only, providing the goodness of fit comparable to the extended hypothesis. This indicates that the spatial hypothesis captures the main pattern in relocations, while the extended hypothesis provides additional insights. Below we describe each of these hypotheses in detail.

### Baseline migration hypothesis: attractiveness potentials

To asses the impact of suburb attractiveness on residential migration flows, we adopt the migration model of Weidlich and Haag^15,40^ which is based on the analogy between the processes of human resettlement and chemical reactions. For each suburb *i* we calculate a value of the potential ${u}_{i}$, which measures the suburb’s attractiveness. The higher the (positive) value of the potential is, the more attractive the suburb is. It can be interpreted as an aggregated indicator which combines all possible characteristics attributed to an individual suburb *i*. These characteristics may include population density, distance to centers of business and retail activity, quality of public services (schools, parks, etc.), transportation connectivity, and other hedonic indicators of the suburb. The notion of the suburb’s potential was introduced in the analysis of inter-regional migration^44^, and its role in the dynamics of residential resettlement has been investigated in our earlier study^40^. In particular, we demonstrated, that the model incorporating the suburb’s potentials contains critical regimes where small fluctuations in the model parameters may result in sharp changes in the spatial population structure. This aspect of the approach is comparable to the Boltzmann-Lotka-Volterra model (also known as Harris and Wilson model) which describes an evolution of the urban structures^13,27^, predicting a qualitatively similar long-term outcome^40^.

In the baseline hypothesis, the migration force ${f}_{ik}$ from suburb *i* to suburb *k* is modelled as

2 $${f}_{ik}={u}_{k}-{u}_{i}.$$

where the sign of the migration force is chosen in such a ways that the migration flows are directed from a suburb with the lower potential to a suburb with the higher potential. Instead of assuming of how the suburb’s potential depends on its characteristics, we *infer their values directly from the data*. In particular, the suburb’s potentials ${u}_{i}$ are treated as independent parameters. For a city with *N* suburbs there are *N*+1 parameters which are fitted to $N(N-\mathrm{1)}$ migration flows between each pair of suburbs. This model allows us to test the hypothesis that the migration flow from suburb *i* to suburb *k* depends only on the difference in individual characteristics of these suburbs (which we call “attractiveness”), and does not depend on other pairwise characteristics (such as distance or other types of proximity). In other words, we test whether the attractiveness ${u}_{k}$ of suburb *k* equally enhances the migration flows ${T}_{ik}$ from all suburbs *i*, regardless of their proximity to *k*.

The results of calibration for the period of 2011–2016, and the corresponding number of model parameters are presented in Table [1] (and in Table [3] in the supplementary material for the 2006–2011; the revealed values of attractiveness are displayed in Fig. 7 in the Supplementary Material). The determination coefficient ${R}^{2}$ is very low, indicating that the attractiveness potentials ${u}_{i}$ fail to explain systematic migration patterns. This in turn means that the impact of any suburb’s characteristic on the residential migration is low and the attractiveness alone does not provide significant information on the resettlement flows.

### Extended migration hypothesis: attractiveness potentials together with relocation distance

We next extend the baseline hypothesis to include additional characteristics which may affect the relocation process. In particular, we assume that the force driving a migration flow from suburb *i* to suburb *k* depends also on the distance ${d}_{ik}$ between these suburbs, so that the migration force ${f}_{ik}$ is set as follows:

3 $${f}_{ik}={u}_{k}-{u}_{i}-\gamma \,{d}_{ik},$$

where $\gamma$ is the same for all suburbs. This model extends the baseline model (2) by adding a pairwise characteristic ${d}_{ik}$, i.e., the distance between the destination *k* and the source *i* of the corresponding migration flow ${T}_{ik}$. In particular, the migration flow to suburb *k* is determined not only by its intrinsic attractiveness, but also by the proximity of a source *i* to the destination *k*.

The factor $\gamma$ can be interpreted as the dissatisfaction associated with the relocation distance, and will be referred to as the relocation impedance. A high value of $\gamma$ means that people relocate mostly to neighbouring suburbs, while a lower value of $\gamma$ means that people relocate both to neighbouring and more distant suburbs with equal preference.

Distance-dependent migration is investigated in various areas of social and biological sciences, such as population biology^45–47^, daily commutes^6^, shopping activity^5,48^, trafficking of goods^6,49,50^, inter-city phone calls^51^. There are various functional forms of the distance dependence. The most common ones are the inverse quadratic^48,50,51^, exponential^13,36,52,53^, their combination^50^ and Gaussian-like^46,54^ functions. We adopt this approach to describe the internal city migration, and use the exponential form of the distance dependence.

**Figure 3:**
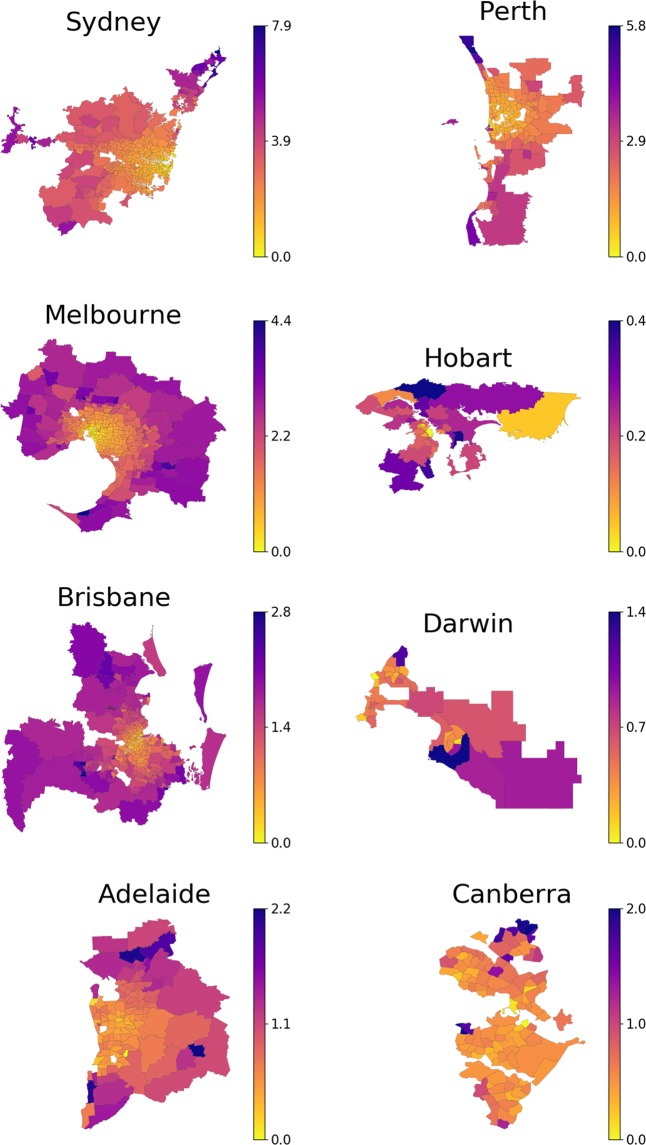
Values of the revealed attractiveness ${u}_{i}$ fitted to 2011–2016 migration data. Potential of each suburb varies from the highest value (blue) to the lowest value (yellow) for each particular GCA. The bar on the right encodes the value of the potentials ${u}_{i}$ whose values are normalized so that minimum value is zero. Note that the density range and therefore the color correspondence are different for each GCA. The suburbs with the population density less than 10 people/km^2^ are not shown.

The results of the calibration include now the value of the relocation impedance, which is listed in Table 2, in addition to the values of the attractiveness of each suburb (Fig. 3). The quality of fit (Table 1) of the extended hypothesis increases substantially in comparison to the baseline hypothesis (2). The values of the coefficient of determination ${R}^{2}$ indicate that contribution of the individual potentials to the quality of the overall fit is much lower than the contribution of the distance between the suburbs. This means, among other things, that the share of relocations which are driven by the attractiveness is much smaller in comparison to the relocations driven by geographic proximity.

**Table 2:** Relocation impedance calibrated from the 2011–2016 data using models (3) and (4). Model (2) essentially implies $\gamma =0$.

GCA	baseline	extended	spatial
Sydney	0	1.01	0.91
Melbourne	0	0.94	0.88
Brisbane	0	0.83	0.79
Adelaide	0	0.82	0.78
Perth	0	0.85	0.76
Hobart	0	0.74	0.73
Darwin	0	0.25	0.24
Canberra	0	0.36	0.36

In addition, we observe that the fitted values of the relocation impedance $\gamma$ reveal a certain pattern. For the majority of the cities the values of $\gamma$ are within the interval 0.75–0.90. In contrast, the relocation impedance values for Canberra and Darwin are within 0.25–0.35, i.e., the range which significantly differs from other capital cities.

Finally, comparing the values of the attractiveness potentials in Fig. 3 with the population density in Fig. 2, we observe a notable correlation between them (as shown in Fig. 8 in the Supplementary Material). In the majority of the cities the densely populated central suburbs appear to be less attractive for residential migration, while the remote suburbs seem to attract a higher number of new residents. In contrast, in Darwin and Canberra, the migration flows are directed to the suburbs around the highly populated areas. These observations are consistent with theoretical studies^15,40^ where it is assumed that the attractiveness potential increases with the population density only until a certain threshold level, beyond which the attractiveness goes down. This means, in particular, that for the majority of Australian capitals this threshold value is quite low. This is fairly surprising, since even the largest Australian capitals have very low population density compared with European and Asian cities which have not reached such threshold yet (cf. 20,781 people/km^2^ in Paris^55^ and 6,158 people/km^2^ in Tokyo^56^ vs the values in Table 4 in the supplementary material in Australian capitals).

It is worth noting that this pattern becomes visible only in the extended model (3), whereas the values of potentials ${u}_{i}$ estimated in accordance with the baseline model (2) are not well defined (Fig. 7 in the Supplementary Material). This demonstrates the importance of including the relocation distance into the migration model for a sensible estimation of ${u}_{i}$.

### Spatial migration hypothesis: relocation distance without attractiveness potentials

In order to contrast the roles of the relocation distance and the attractiveness potentials, we may consider a simplification of the extended hypothesis, by assuming that the migration force ${f}_{ik}$ is determined by only the relocation distance:

4 $${f}_{ik}=-\,\gamma \,{d}_{ik}.$$

In this way we abstract away a large number of parameters (the attractiveness of each individual suburb), and keep only two parameters: the relocation impedance and the normalization factor.

The calibrated values of the relocation distance are shown in Table 2,while the quality of fit is shown in Table 1 (and Table 3 in the supplementary material). The calibrated values of $\gamma$ for the spatial model are similar to the ones for the extended model (3). Canberra and Darwin substantially differ from the other capitals by the values of relocation impedance having magnitude in the range of 0.22–0.24 (Darwin) and 0.34–0.36 (Canberra), compared to 0.73–0.94 in the other cities. Despite a significant reduction in the number of model parameters (from “number of suburbs” plus two in (3) to just two in (4)), the goodness of fit has not changed substantially (${R}^{2}$ decreased by 10–15 percentage points).

### Relating migration patterns to spatial structure of cities

The presented results lead to two important conclusions. Firstly, we observe that temporal stability in intra-urban migration flows (Fig. 6 in the Supplementary Material) can be explained by the fact that the most intensive migration occur between suburbs that are geographically close, while the individual characteristics of suburbs have a significantly smaller influence on the residential migration in the majority of GCA. Secondly, there are two capital cities whose migration patterns substantially differ from those shown by the other capitals. In particular, in Darwin and Canberra, the suburbs’ characteristics (represented by the attractiveness potentials) play more important role in shaping the residential migration, and the long distance relocations occur more often than in other capital cities. In this section we investigate how the spatial city structure affects these relocation patterns.

First, we note that canonical properties used to characterize cities, such as the population or the area (Fig. 1), do not explain the observed difference in the migration patterns, as evidenced by Figs. 10 and 11 in the supplementary materials. In particular, Hobart has low population and area but displays a high relocation impedance. This contrast is also observed in small non-capital cities with a sufficiently representative number of suburbs. Furthermore, the overall variation in the population and area of the capital cities does not concur with the variation of the relocation impedance. This indicates that the relocation impedance may depend on other, more refined, characteristics of a city which better reflect its internal structure.

There exists a number of approaches which identify the most important characteristics of urban spatial structures, including compactness and spreading^10,57^, spatial clustering of hotspots (areas with high population density or intensive economic activity)^58^, monocentricty versus polycentricity^59^, population density profile^60^, etc. In a recent study^10^, two main indicators capturing the whole diversity of the possible urban structures have been introduced: the degree of heterogeneity and the degree of spreading. Here we adopt the latter, and investigate to what extent the suburbs with a high population density are localized together, and how far they are spread across a city.

The spreading index^10^ is defined as the ratio of the average distance between the highly populated areas to the average distance between all areas in a city:

5 $$\eta (\rho )\,=\,\frac{\sum _{i,j}\,{d}_{ij}\,{\xi }_{i}(\rho )\,{\xi }_{j}(\rho )}{\sum _{i,j}\,{d}_{ij}{S}_{i}{S}_{j}}$$

where ${\xi }_{i}(\rho )\equiv {S}_{i}\Theta ({\rho }_{i}-\rho )\,S/S(\rho )$, with $\Theta (x)$ being the Heaviside step function, ${S}_{i}$ is the area of suburb *i*, *S* is the total area of suburbs, and $S(\rho )$ is the area of suburbs with the population density higher than $\rho$. The spreading index is a function of the density, declining rapidly from 1 to 0 if the city is monocentric, in which case the most populated suburbs are heavily clustered within a single area. In contrast, if the suburbs with high population density are spread all around the city, $\eta (\rho )$ remains high within a large range of $\rho$ values. It is possible to evaluate a single value of the spreading index ${\eta }^{\ast }$ (instead of the entire function $\eta (\rho )$), for a particular value of the threshold density ${\rho }^{\ast }$, reflecting the intuitive concepts of city “spreadness” and “monocetricity”^10,61^. To evaluate particular threshold values of the density ${\rho }^{\ast }$ for each GCA, as well as the spreading index ${\eta }^{\ast }=\eta ({\rho }^{\ast })$, we follow methods quantifying spatial urban organization with respect to the population density^10,61^. The threshold densities for each GCA are listed in Table 4 of the supplementary material.

**Figure 4:**
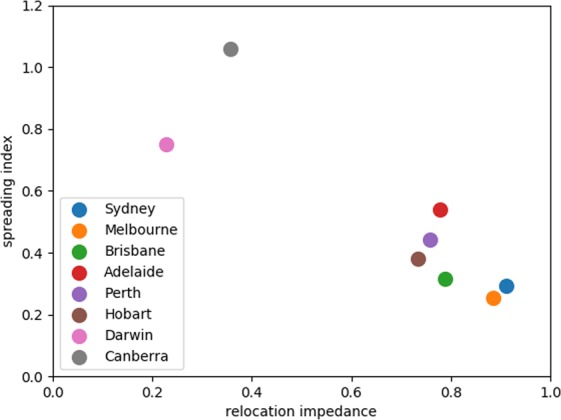
Resettlement diagram for Australian capital cities. The vertical axis measures the spreading index (5) at a threshold population density (Table 4 in the supplementary material), while the horizontal axis measures the city’s relocation impedance of the spatial model ((4), Table 2). Each GCA corresponds to a point on the diagram.

The spreading index ${\eta }^{\ast }$ is plotted against the corresponding relocation impedance $\gamma$ in Fig. 4. In cities with a low spreading index we observe a higher relocation impedance (which is typical for compact monocentric cities^10^). Conversely, a low relocation impedance is observed in cities with high ${\eta }^{\ast }$ (which is typical for spread-out polycentric cities^10^). We can identify the cluster of monocentric cities with a high relocation impedance, for which $\gamma$ is in the interval $0.8\pm 0.12$, while ${\eta }^{\ast }$ is in the interval $0.4\pm 0.14$. This cluster is robust with respect to an extension to non-capital cities with comparable population and the number of suburbs. In particular, the smallest number of suburbs among all GCAs is 35 (Hobart).

Most of non-capital Australian cities have less than 30 suburbs, with only two exceptions (Gold Coast and Sunshine Coast). The relocation impedance values for Gold Coast and Sunshine Coast are 0.68 and 0.81 respectively (not shown in the figure), and the corresponding values of spreading index are 0.51 and 0.41. This means that these two cities belong to the monocentric cluster with the high relocation impedance as well.

**Figure 5:**
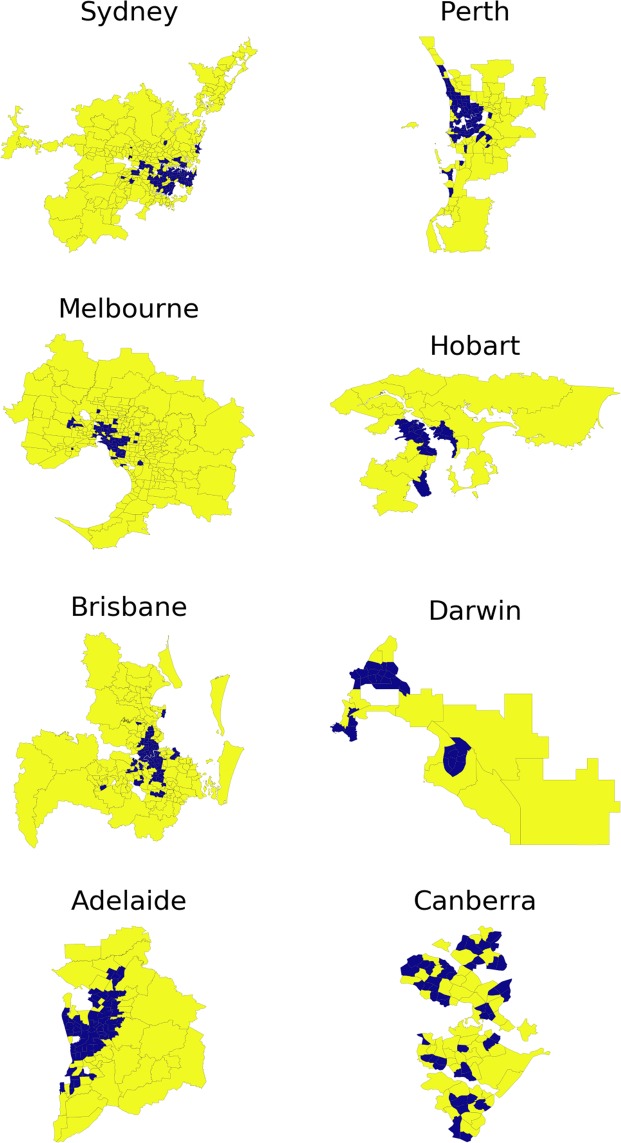
Binary density map of GCA for 2016 Census. The population density of each suburb is compared to the threshold density calculated by the LouBar methodology^10,61^ (see Table 4 in the Supplementary Material) of the corresponding city, and is identified as being high (blue) or low (yellow). The suburbs with the population density less than 10 people/km^2^ are not shown.

The spreading index analysis is illustrated in Fig. 5: the population density of each suburb is compared to the threshold density calculated by the LouBar methodology^10,61^ (see Table 4 in the supplementary material) of the corresponding city, and is identified as being high (blue) or low (yellow). We observe that a clear majority of the cities (Sydney, Melbourne, Brisbane, Adelaide, Perth, Hobart) include high-density suburbs, predominantly clustered around a single center. In contrast, the two remaining cities (Darwin and Canberra) include high-density suburbs clustered around multiple centers. Comparing Fig. 5 with the actual density distribution within each GCA in Fig. 2, we conclude that the adopted method (comprising the binary threshold ${\rho }^{\ast }$ and spreading index ${\eta }^{\ast }$) indeed reflects the intuitive perception of a city “spreadness”.

The spreading index reflects structural properties of a city, while the relocation impedance reflects its dynamic properties. The observed correlation between these two quantities suggests that the population dynamics is likely to be influenced by urban spatial structure.

## Conclusions

Our analysis of intra-urban resettlement in the Australian Capital Cities leads to several important conclusions. In particular, we observe that the revealed attractiveness of individual suburbs (which may include, among other characteristics, the distance to the city center, population density, quality of infrastructure, etc.) alone cannot explain the migration flows with a satisfactory precision. In contrast, we discover that the relocation distance alone provides much higher determination of migration flows than the suburbs’ attractiveness.

It is interesting to note that while the importance of the geographical distance has been shown for various types of migration (in particular, inter-city migration^6,62^, intra-urban commutes^13,52^, or even migration of animals^63–65^, which obey the so-called “gravity law”), it is generally believed that the relocation distance is not important for intra-urban resettlement. In particular, the traditional understanding of how the distance affects migration relies on the associated transportation costs^62^, which are supposed to be negligible for relocations within the same city. Here we show that this is not the case for resettlement in Australian capital cities, and that the relocation distance alone can explain circa 50% of the variation in migration flows. Moreover, we observe that all capital cities except for Darwin and Canberra form a cluster of monocentric cities with high relocation impedance. In particular, we see that with respect to intra-urban resettlement, the cities which differ in size, population, transportation network and other dynamical details, reveal similar migration patterns which are independent of these features.

In addition, we observe that the spatial structure of the attractiveness revealed from the migration data is opposite to the static settlement patterns with central districts being more popular than the city outskirts. Instead we show that the most attractive suburbs are located not in the central areas with high population density and intensive business and retail activity, but in sparsely populated outskirts. These results are also consistent with observations typically reported by various real estate analysts^66^.

We report two exceptions to these more general observations: Darwin and Canberra. In particular, in these GCAs i) the migration flows are directed towards more densely populated areas; ii) the relocation impedance is low; iii) the spreading index is high. This indicates that the migration dynamics in these cities is significantly different from the one across all other GCAs. While this can be simply a coincidental feature, we speculate that this is a consequence of historical development of these cities, in which centralized governmental planing played a much larger role than in the other GCAs which followed more than 100 years of essentially self-organized evolution. Indeed, a large part of Canberra was built in the period during 1961–1981^67^, in accordance with government housing and developing programs^68^, while Darwin was completely rebuilt twice: after WWII, and in late 1970s following Cyclon Tracy which destroyed 80% of houses^69,70^. We believe that this can be an indication of the differences between self-organized and planned development trajectories of cities. It would be interesting to verify such a conjecture for other cities which were highly influenced by centralized planning (e.g., Brasilia, Cancun, Singapore, Washington D.C., etc.).

## Materials and Methods

To perform our analysis, we use the data about internal migration in eight Australian capital cities (Sydney, Melbourne, Brisbane, Adelaide, Perth, Hobart, Darwin and Canberra) from the Australian Census datasets (2011 and 2016), provided by the Australian Bureau of Statistics^35^. We analyze the Greater Capital Areas (GCAs), which represent urbanized contiguous areas around the corresponding cities. The Census data are organized by the so-called “statistical areas” (SAs), where people reside. The data also contain information on where people resided at the Census date and 5 years before the Census date. The information is provided on the aggregate level in terms of the number of people, and not on the individual level. The data are available in various resolutions, and we use the “Statistical Areas Level 2” (SA2s) resolution. There are 2,310 SA2 regions in entire Australia and between 30–300 SA2s in each GCA. Each SA2 has a population generally within a range of 3,000–25,000 persons^35^, while some of SA2s within each GCA, such as national parks or industrial objects, have negligible population. In our analysis, we discard suburbs with the population density less than 10 persons/km^2^, setting the difference between the urban and the rural suburbs. This particular value is chosen to indicate a threshold beyond which the spreading index ${\eta }^{\ast }$ becomes insensitive to the threshold variation, as shown in Fig. 9 of Supplementary Material.

We built the dataset of migration flows, which makes a correspondence between each pair of statistical areas $(i,k)$ and the amount of people relocated from *i* to *k*. The values of ${d}_{ik}$ are calculated as distances between centroids of suburbs *i* and *k*. To take into account the fact that the sizes of suburbs are not uniform (and hence, distances between big suburbs are larger), we normalize ${d}_{ik}$ by factor ${({S}_{i}{S}_{k})}^{-\mathrm{1/4}}$ where ${S}_{i}$ and ${S}_{k}$ are the areas of suburb *i* and *k* respectively. This means that ${d}_{ik}$ is a dimensionless quantity, measured in the number of suburbs (as linear measurements of a suburb are proportional to the square root of its size).

In our analysis we do not consider people who do not change their place of residence within the observation period (the case when $i=k$) because their number is significantly larger than the number of those who do, and it might distort the model parameters’ estimates. Moreover, it is reasonable to expect that quantities ${T}_{ii}$ cannot be described by the same model because the magnitude of migration flow ${T}_{ik}$ ($i\ne k$) goes up as the time of observation increases, while the number of people ${T}_{ii}$ staying in the same area within this period goes down. Also, we do not analyze external migration flows (in and out of GCA).

The fitting procedure is performed numerically using the ordinary least square technique and the trust region reflective algorithm implemented in SciPy 1.3.0 library in Python^71^ (method scipy.optimize.leastsq): $\theta ={\rm{\arg }}{{\rm{\min }}}_{{\rm{\theta }}}{\sum }_{i,j}{({T}_{ij}-{\hat{T}}_{ij}(\theta ))}^{2},$ where $\theta$ is a vector of parameters that include $\mu$ and potentials ${u}_{i}$ in model (2), $\mu$, $\gamma$ and potentials ${u}_{i}$ in model (3), $\mu$ and $\gamma$ in model (4), where ${T}_{ij}$ is actual migration flow from suburb *i* to suburb *j*, and ${\hat{T}}_{ij}(\theta )$ is the migration flow calculated according to the corresponding model for the parameters given by vector $\theta$. Note that despite the form suggested by Eq. (1), minimizing the logarithmic residual is not feasible, as there exist many suburb pairs with zero migration between them.

## Supplementary information

Supplementary Material.

## Data Availability

All data needed to evaluate the conclusions in the paper are available from Australian Bureau of Statistics^35^.
