# Supplementary material for: City structure shapes directional resettlement flows in Australia
Source: Sci Rep. 2020 May 19;10:8235. doi: 10.1038/s41598-020-65208-5 (PMC7237677; doi:10.1038/s41598-020-65208-5)
Supplement: Supplementary file 1 — Supplementary Material. [file 41598_2020_65208_MOESM1_ESM.pdf]

City structure shapes directional resettlement flows in Australia

Bohdan Slavko, Kirill Glavatskiy, Mikhail Prokopenko

Migration flows: inter-temporal comparison

Intra-urban migration flows between each pair of suburbs within 2006-2011 plotted against the corresponding flows within 2011-2016 are shown in Fig. 6.

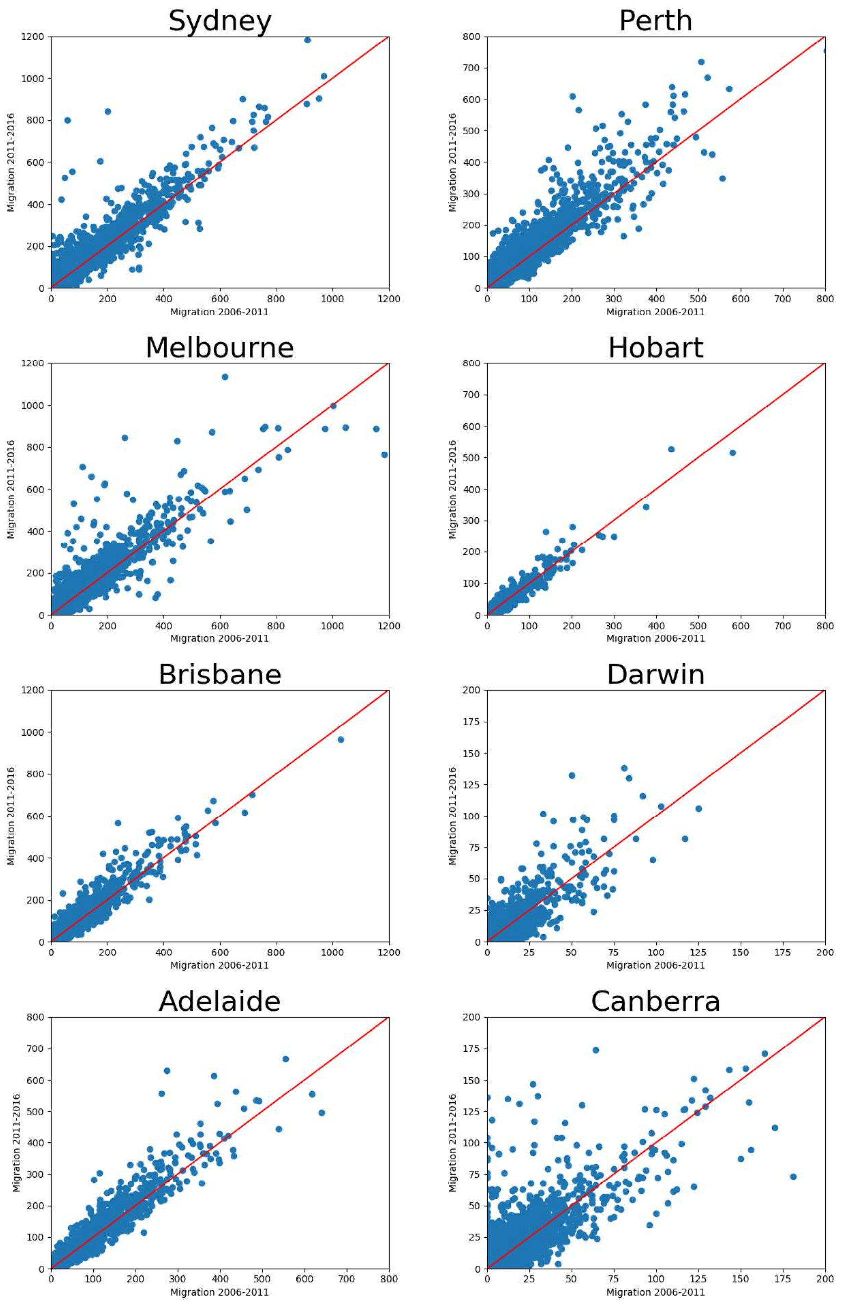

Figure 6. Residential migration in the Australian capital cities. Intra-urban migration flows between each pair of suburbs within 2006-2011 plotted against the corresponding flows within 2011-2016. Solid red line corresponds to the set of points where both flows are equal.

Attractiveness potentials of suburbs

The values of revealed attractiveness estimated according to model (2) are shown in Fig. 7.

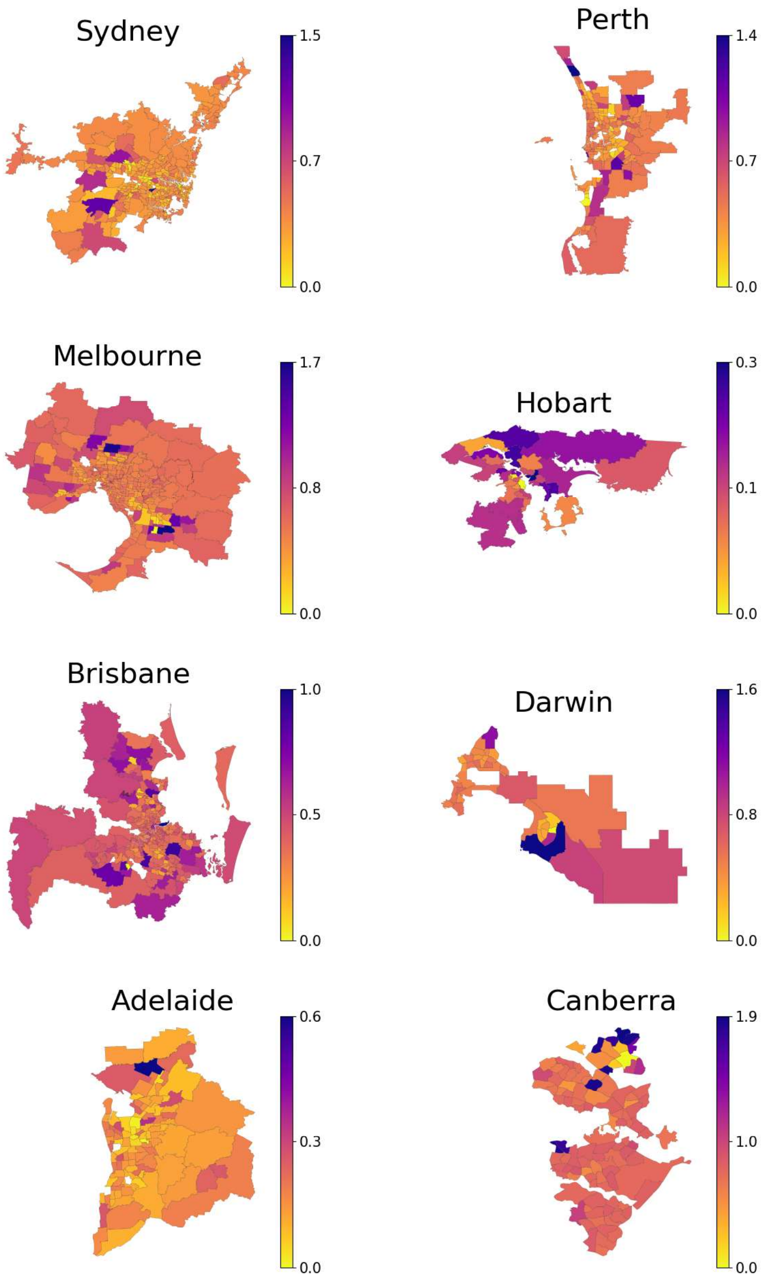

Figure 7. Values of the revealed attractiveness u_i (model (2)) fitted to 2011–2016 migration data. Potential of each suburb varies from the highest value (blue) to the lowest value (yellow) for each particular GCA. The bar on the right encodes the value of the potentials u_i whose values are normalized so that minimum value is zero. Note that the density range and therefore the color correspondence are different for each GCA. The suburbs with the population density less than 10 people/km^2 are not shown.

Correlation between revealed attractiveness and population density

The values of revealed attractiveness (model (3)) plotted against population density in the corresponding areas are shown in Fig. 8.

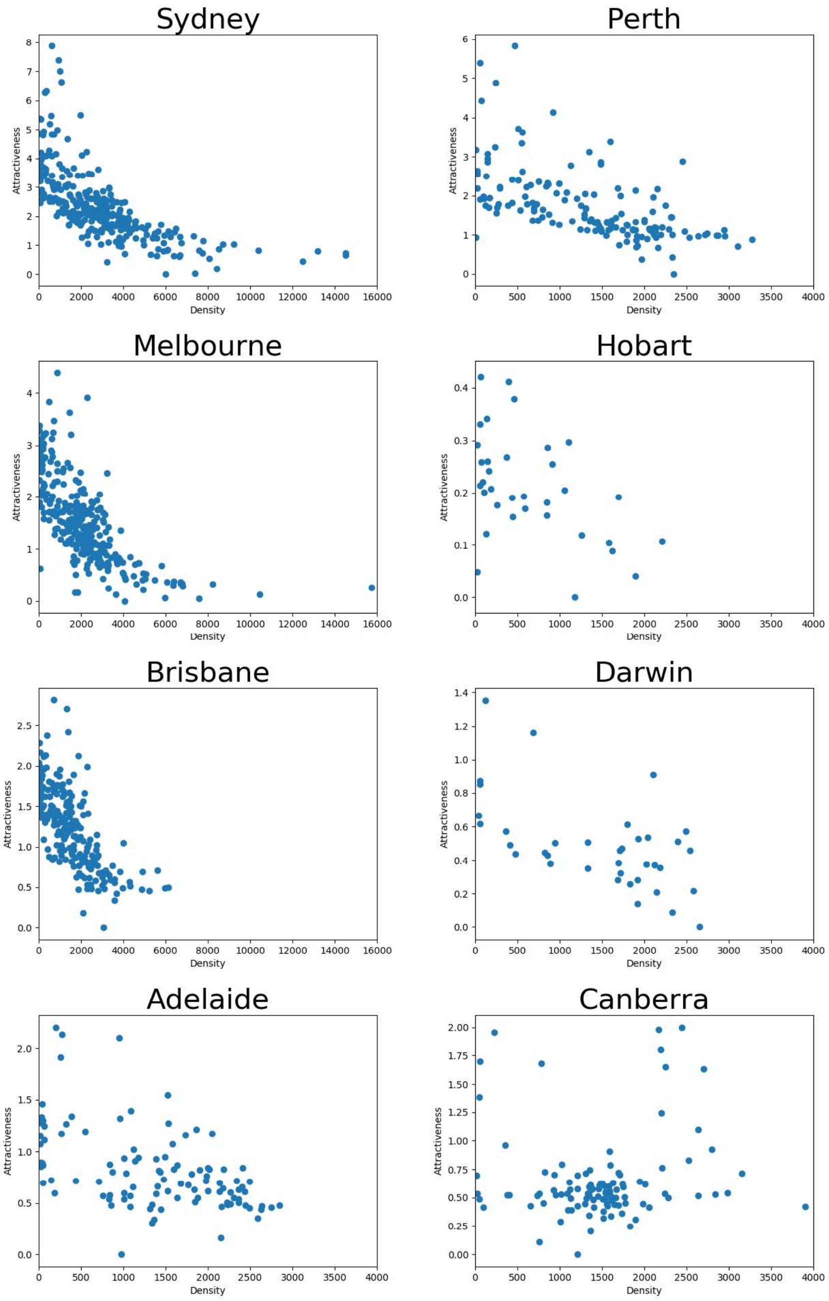

Figure 8. Values of attractiveness potentials u_i (vertical axis) fitted to 2011–2016 migration data (model (3)) plotted against population density level (horizontal axis). Values of u_i potentials are normalized so that minimum value is 0.

Calibration to 2006–2011 data

Results of the model calibration to the 2006–2011 data are shown in Table [3].

Table 3. Relocation impedance γ and coefficient of determination R^2 for migration models (2)-(4) fitted to the 2006–2011 data

GCA	γ			R^2		
	model (2)	model (3)	model (4)	model (2)	model (3)	model (4)
Sydney	0	1.04	0.94	0.04	0.68	0.61
Melbourne	0	1.02	0.92	0.04	0.71	0.56
Brisbane	0	0.87	0.80	0.06	0.70	0.63
Adelaide	0	0.84	0.80	0.12	0.75	0.69
Perth	0	0.89	0.81	0.12	0.72	0.58
Hobart	0	0.72	0.74	0.16	0.67	0.64
Darwin	0	0.22	0.22	0.40	0.66	0.57
Canberra	0	0.37	0.34	0.30	0.76	0.61

GCA densities

The information about GCA population density in shown in Table [4].

Table 4. Threshold population density ρ^* calculated by the LouBar methodology[10,61] for each GCA, compared to the average population density $\overline{\rho}$ (excluding suburbs with the population density less than 10 persons), from the 2016 Census data.

GCA	ρ^*, people/km^2	$\overline{\rho}$, people/km^2
Sydney	3 455	846
Melbourne	2 828	451
Brisbane	1 656	316
Adelaide	1 135	407
Perth	1 306	465
Hobart	446	138
Darwin	855	279
Canberra	1 533	1 204

GCA spreading index plotted for different values of the population density cut-off

Spreading index values η^* plotted against population density density cut-off (suburbs with density below this threshold are excluded from the index calculation) are shown in figure Fig. 9.

Relocation impedance versus population and area

Relocation impedance, γ (model (4)), plotted against population and area of the largest Australian cities is shown in Fig. 10 and Fig. 11 respectively. In the group of cities with high relocation impedance (including Sydney, Melbourne, Brisbane, Adelaide, Perth, Hobart) population varies from 222 345 people in Hobart to 4 823 984 in Sydney (22 times bigger) and area varies from 1 695 km^2 in Hobart to 15 841 km^2 in Brisbane (10 times bigger) while relocation impedance of the spatial model (4) changes insignificantly from 0.73 to 0.91. Moreover, non-capital cities with comparable population and number of suburbs (the smallest amount of suburbs among all GCAs is 35, and is observed in Hobart; most of non-capital Australian cities have less than 30 suburbs). Gold Coast (population: 569 988, area: 1 858 km^2, number of suburbs: 48) and Sunshine Coast (population: 346 532, area: 3 085 km^2, number of suburbs: 33), exhibit relocation impedance of 0.68 and 0.81 respectively which is close to the values observed in the group of capital cities with high relocation impedance (which includes Sydney and other big cities). At the same time Darwin's and Canberra's population and area fall within the corresponding first group's intervals, their relocation impedance values drastically differ from the first group: $\gamma = 0.24$ in Darwin and $\gamma = 0.36$ in Canberra. For this reason, we have to look for another explanation and try to attribute this distinction to other indicators of spatial structure of cities.

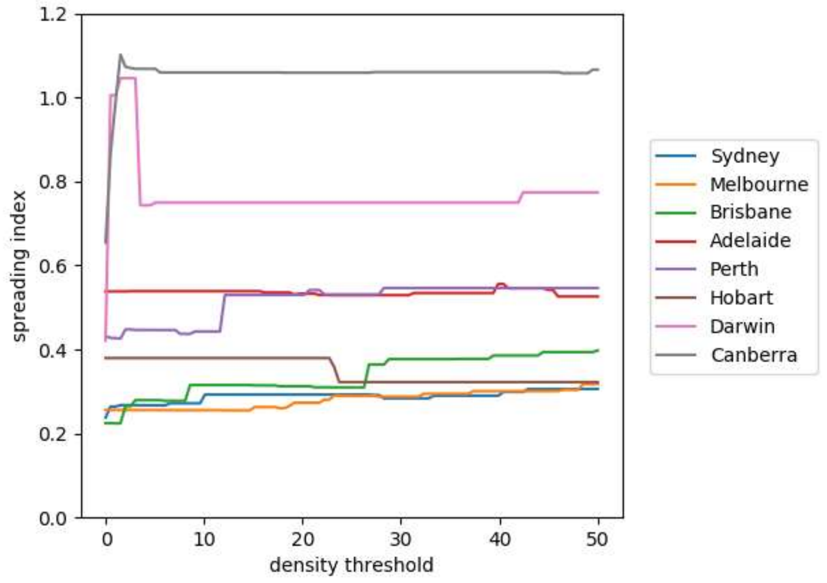

Figure 9. Spreading index η^* of the Australian capital cities plotted against density cutoff threshold (suburbs with density below this threshold are excluded from the index calculation). Each GCA corresponds to a line on the diagram.

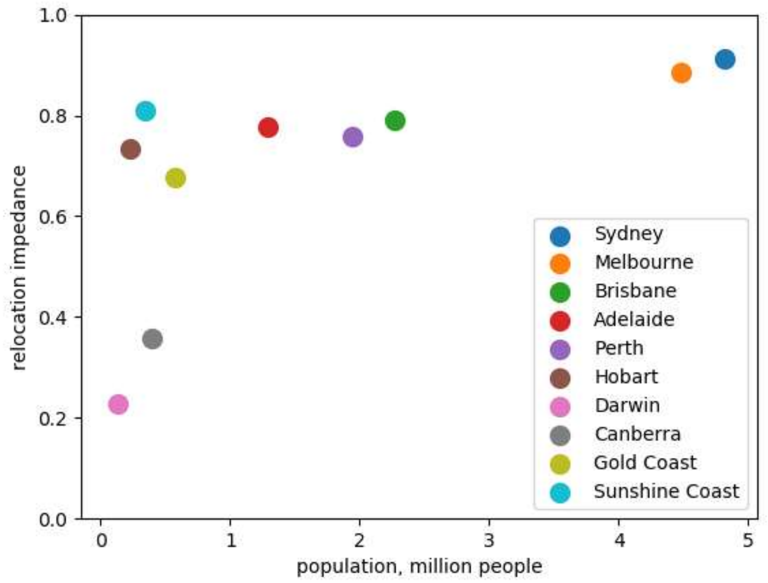

Figure 10. Relocation impedance of the extended list of Australian cities (capital cities + Gold Coast ans Sunshine Coast) plotted against the city population.

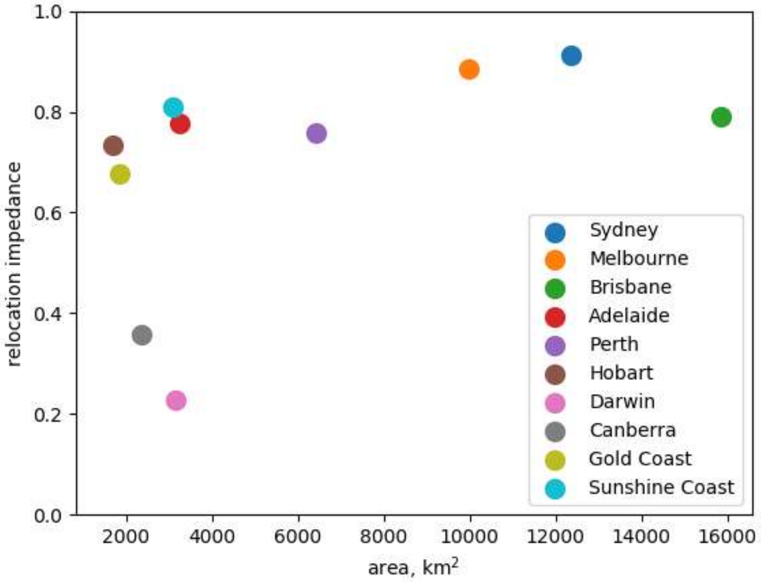

Figure 11. Relocation impedance of the extended list of Australian cities (capital cities + Gold Coast ans Sunshine Coast) plotted against the city area.
